# Prognostic value of ventricular longitudinal strain in patients undergoing transcatheter aortic valve replacement: A systematic review and meta-analysis

**DOI:** 10.3389/fcvm.2022.965440

**Published:** 2022-08-24

**Authors:** Yangjie Xiao, Wenjing Bi, Wei Qiao, Xin Wang, Ying Li, Weidong Ren

**Affiliations:** Department of Ultrasound, Shengjing Hospital of China Medical University, Shenyang, China

**Keywords:** strain, echocardiography, aortic stenosis, transcatheter aortic valve replacement, meta-analysis

## Abstract

**Introduction:**

Strain obtained by speckle tracking echocardiography (STE) can detect subclinical myocardial impairment due to myocardial fibrosis (MF) and is considered a prognostic marker. Aortic stenosis (AS) is not only a valve disease, but also a cardiomyopathy characterized by MF. The purpose of this study was to systematically review and analyze ventricular strain as a predictor of adverse outcomes in patients with AS undergoing transcatheter aortic valve replacement (TAVR).

**Methods:**

PubMed, Embase, and the Cochrane library were searched for studies that investigated the prognostic value of impaired ventricular strain on patients with AS undergoing TAVR with all-cause mortality (ACM) and major adverse cardiovascular events (MACE). Pooled odds ratios (ORs), hazard ratios (HRs), and 95% confidence intervals (CIs) were calculated to assess the role of left (LVLS) and right (RVLS) ventricular longitudinal strain in the prognostic prediction of patients with AS undergoing TAVR. Sensitivity and subgroup analysis was performed to assess heterogeneity.

**Results:**

Twelve studies were retrieved from 571 citations for analysis. In total, 1,489 patients with a mean age of 82 years and follow-up periods varying between 1 year and 8.5 years were included. Meta-analysis showed the impaired LVLS from eight studies was associated with an increased risk for combined ACM and MACE (OR: 1.08, 95% CI: 1–1.16; *p* = 0.037), and ACM alone (HR: 1.08, 95% CI: 1.01–1.16; *p* = 0.032). Impaired RVLS from four studies was associated with an increased risk of combined ACM and MACE (OR: 1.08, 95% CI: 1.02–1.14; *p* < 0.01), and ACM alone (HR: 1.07, 95% CI: 1.02–1.12; *p* < 0.01).

**Conclusions:**

This meta-analysis demonstrated that ventricular strain, including LVLS and RVLS, had a substantial prognostic value in ACM or combined ACM and MACE, which could be used as a valid marker for risk stratification in patients with AS undergoing TAVR.

## Introduction

Aortic stenosis (AS) is the most common valvular heart disease and is characterized by progressive calcification of the aortic valve, obstructing the left ventricular outflow tract, leading to heart failure and even death (1). Heart failure develops from myocardial cell hypertrophy leading to atrophy, myocardial fibrosis (MF), and death (2). Therefore, AS is also considered cardiomyopathy characterized by MF resulting from chronic pressure overload of the left ventricle (3, 4). Aortic valve (AV) replacement to treat AS can be accomplished using surgical and transcatheter approaches. Transcatheter aortic valve replacement (TAVR) has developed as a breakthrough therapeutic advance in the treatment of symptomatic patients with severe AS, especially elderly and vulnerable patients. Recent evidence suggests that TAVR is also a safe option for patients of low to intermediate risk (5, 6). Therapeutic decisions are based on the pre-procedural evaluation, but pre-procedural risk assessment is challenging and data regarding parameters predicting clinical outcomes are limited.

Echocardiography is the primary modality for diagnosis of AS, allowing for evaluation of AV status, including morphology, area, and transvalvular velocities or gradients, which can also be used for assessment of ventricular morphology and function. Measurement of left ventricular (LV) systolic function with LV ejection fraction (EF) has been linked to worse outcomes in patients undergoing TAVR. Speckle-tracking echocardiography (STE) has emerged as a sensitive technique to detect subclinical myocardial impairment due to MF (7, 8). Strain derived from STE is a novel parameter to assess segmental myocardial deformation, which is more sensitive than LVEF in evaluating ventricular dysfunction caused by MF (9) and provides incremental prognostic information in severe patients with AS (10). The LV longitudinal strain (LVLS) assessment provides independent prognostic value and is recommended for inclusion in TAVR risk stratification models (11). The right ventricular longitudinal strain (RVLS) has also been considered a feasible parameter for assessing right ventricular (RV) systolic function and is associated with all-cause mortality (12). However, there is only limited data on the prognostic value of LVLS and RVLS in patients with AS undergoing TAVR and some of the results are conflicting. The purpose of this study was to systematically review and analyze LVLS and RVLS as a predictor of adverse outcomes in patients with AS undergoing TAVR.

## Methods

### Screening of publications

This systematic review and meta-analysis were performed in accordance with the Preferred Reporting Items for Systematic reviews and Meta-Analyses (PRISMA) statement and protocols. Using the electronic databases of PubMed, Embase, and the Cochrane library, publications on patients with AS having taken STE examination undergoing TAVR were searched from the earliest available date of indexing up to March 31, 2022. A search strategy was used based on combined terms: (1) “transcatheter aortic valve replacement” or “TAVR” or “transcatheter aortic valve implantation” or “TAVI” and (2) “speckle tracking” or “strain” or “STE” and (3) “echocardiography”, or “echocardiogram” and (4) “ventricular” or “ventricle.” Ethics committee approval was not necessary because all data was extracted from existing literature.

### Data extraction and quality assessment

According to the above protocol, duplicate records and studies that did not provide information of interest were excluded. The parameter LVLS or RVLS was evaluated by STE in patients with AS undergoing TAVI, with sufficient data to retrieve odds ratio (OR), hazard ratio (HR), and corresponding 95% confidence intervals (CIs). The primary endpoint was all-cause mortality (ACM). The secondary endpoint was major adverse cardiovascular events (MACE) comprising cardiovascular death and any cardiovascular events, including hospital admission for heart failure, acute myocardial infarction, or stroke. Data on demographic variables and echocardiographic parameters were also extracted from each study. Demographic variables included sample size, mean age, gender, body weight index (BMI), history of hypertension, diabetes mellitus (DM), dyslipidemia, coronary artery disease (CAD) and chronic obstructive pulmonary disease (COPD), mean STS score, NYHA functional class, risk profile, the mean or median time of follow-up, and hospitalization rate and event rate. Echocardiographic parameters included mean AV area, mean AV pressure gradient, AV velocities, LVEF, and LVLS or RVLS. Two researchers independently reviewed selected articles and when there was disagreement between authors, consensus on final inclusion was reached through a third researcher.

The quality of included studies was assessed using the Newcastle–Ottawa quality assessment scale (NOS) in three broad categories. The scores were displayed on a nine-point scale with poor quality (0–2 points), medium quality (3–5 points), and high quality (6–9 points).

### Data synthesis and statistical analysis

The OR and HR with 95% CIs were extracted for meta-analysis. If the studies did not report HRs directly, Kaplan–Meier curves were read using Engauge Digitizer version 4.1. The pooled effect was evaluated using *Z* scores. Heterogeneity among studies was assessed using Chi-square Cochran's *Q* test to measure the inconsistency. The *I*^2^ statistic was used to describe the proportion of total variation in studies due to heterogeneity. An *I*^2^ statistic of <25% indicated low heterogeneity, while over 50% indicated a high heterogeneity. Statistical analyses were performed using STATA V.15.1 (Stata Corp LP), with *p* < 0.05 considered statistically significant. Publication bias was assessed by the Egger's test for included studies. Sensitivity analyses were performed by one-to-one exclusion studies to estimate the stability of pooled results.

## Results

### Literature search and study selection

**Figure 1:**
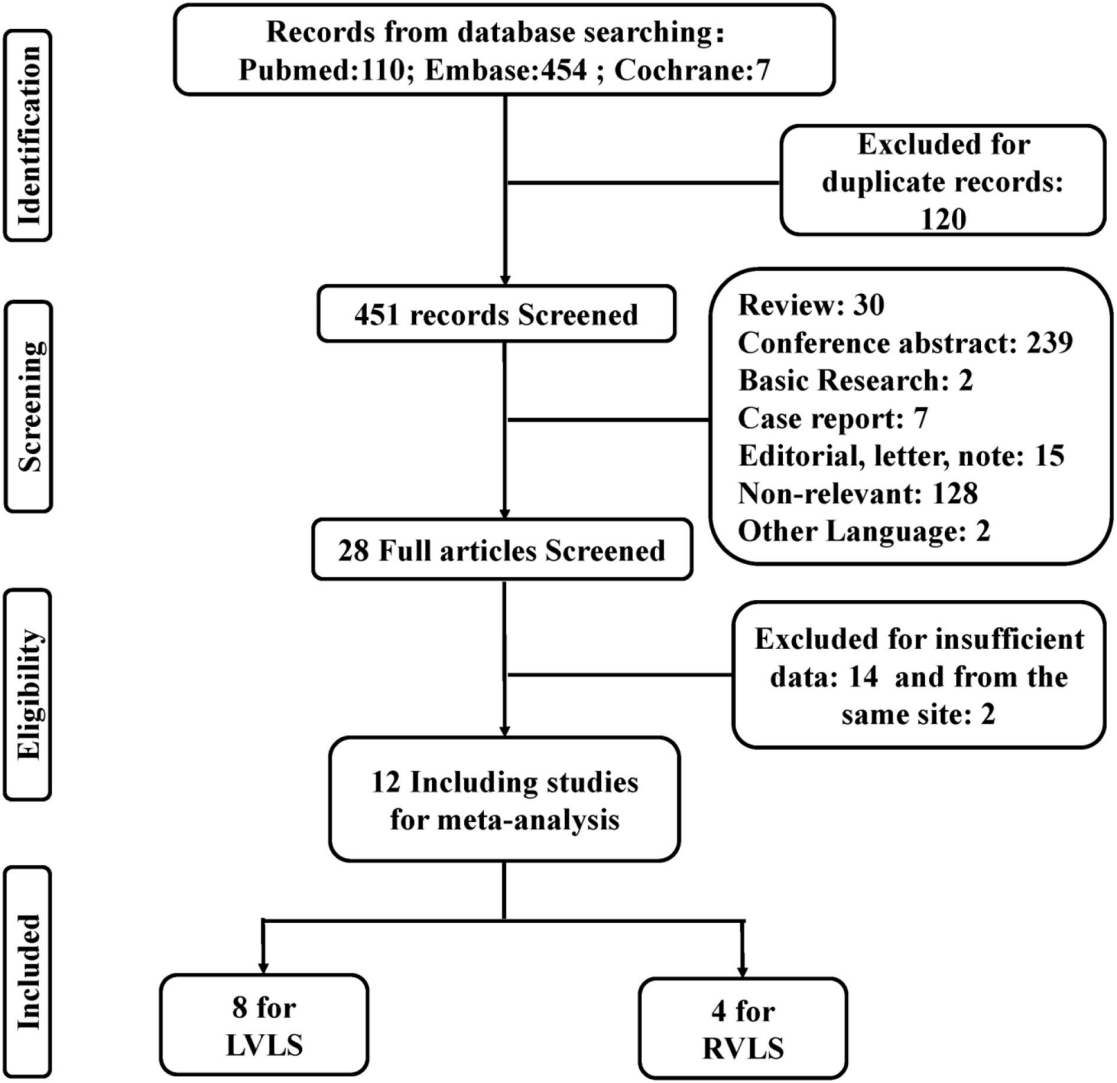
Flow diagram of literature inclusion.

A total of 571 records were found from the electronic databases using the search strategy, with 120 duplicate records excluded. Articles without data of interest providing useful data were excluded, including 239 conference abstracts, 30 reviews, two basic research studies, seven case reports, 15 editorials, notes and surveys, 128 non-relevant records, and two non-English language studies. The remaining 28 studies were further evaluated based on full-text articles. Another 14 articles were excluded due to insufficient data and two articles were eliminated because of overlapping data from the same site (13, 14). The remaining 12 studies were included in the meta-analysis to calculate pooled OR or HR, two of which were read using Engauge Digitizer (11, 15). Of these, eight were used for LVLS analysis and four for RVLS analysis. The study selection procedure is shown in Figure 1.

### Study characteristics and quality assessment

A total of 12 studies involving 1,489 patients were included for final analysis, of which two were prospective and 10 were retrospective. The size of the patient population varied significantly between 88 and 499 individuals, of which 44.4% were male and the mean age was 82 years old. Eighty-two percent of patients had hypertension, 31% had diabetes mellitus, 76% had dyslipidemia, 56% had CAD, 18% with COPD, 69% NYHA functional class III/IV, and the mean STS score was seven. Most patients were at high-risk for operation. These studies were published between 2017 and 2022. The characteristics of the studies and the participants are summarized in Table 1. All the included studies were high quality using NOS, with six studies receiving eight points and six studies receiving seven points, as presented in Table 1.

**Table 1:** Characteristics of included studies.

**Study**	**Country**	**Year**	**Design**	**Size**	**Male**	**Mean age (years)**	**Mean BMI (kg/m^2^)**	**Hypertension**	**Diabetes mellitus**	**Dyslipidemia**	**CAD**	**COPD**	**NYHA functional class III/IV**	**Mean STS score**	**Risk profile**	NOS*
Wani et al. (16)	USA	2022	Retrospective	204	46.0%	85	NR	71.6%	32.8%	NR	69.1%	36.3%	80.0%	6.9	High	7
Shimoni et al. (17)	Israel	2021	Prospectively	88	44.3%	81	28.6	94.0%	43.0%	91.0%	60.0%	NR	15.0%	NR	NR	8
Ferreira et al. (18)	Portugal	2021	Retrospective	89	43.8%	82.1	27.1	86.5%	28.1%	64.0%	51.7%	NR	71.9%	5.5	NR	7
Omran et al. (19)	Germany	2021	Retrospective	229	38.0%	83.8	NR	78.6%	21.0%	NR	54.1%	15.7%	84.3%	5.6	High	8
Koschutnik et al. (20)	Austria	2021	Prospectively	204	49.0%	80.9	26.9	90.0%	28.0%	69.0%	53.0%	11.0%	65.0%	3.8	NR	7
Vizzardi et al. (21)	Italy	2020	Retrospective	56	42.9%	81.6	26.6	69.6%	28.6%	NR	50.0%	21.4%	75.0%	NR	High	8
Medvedofsky et al. (12)	USA	2020	Retrospective	334	41.0%	83	27	94.0%	33.0%	79.0%	58.0%	11.0%	88.0%	9.2	High	7
Dahl Pedersen et al. (22)	Denmark	2020	Retrospective	499	47.0%	79.8	NR	75.0%	21.0%	NR	29.0%	14.0%	65.0%	NR	NR	8
Fukui et al. (11)	USA	2020	Retrospective	331	49.0%	83	NR	NR	NR	NR	NR	NR	NR	8.4	High	8
Suzuki-Eguchi et al. (23)	Japan	2018	Retrospective	128	16.0%	83.7	NR	72.7%	27.3%	NR	34.4%	NR	NR	NR	NR	7
Sato et al. (28)	USA	2017	Retrospective	209	58.0%	81	NR	84.0%	41.0%	78.0%	84.0%	NR	94.0%	9.6	High (69%)	8
Kobayashi et al. (15)	USA	2017	Prospectively	128	58.0%	83.4	27.1	84.0%	32.0%	72.0%	75.0%	NR	56.0%	7.8	High	7

BMI, body mass index; CAD, coronary artery disease; COPD, chronic obstructive pulmonary disease; STS, Society of Thoracic Surgeons; NYHA, New York Heart Association; NOS, Newcastle-Ottawa Scale; NR, Not reported.

*The median/mean follow-up time ≥24 months were scored.

Echocardiographic parameters of the included studies presented with a mean AV area of.64 cm^2^ and a mean pressure gradient ranging from 41 to 57 mm Hg. The average LVEF was 57%. The mean pre-TAVR LVLS was −14% and RVLS was −21%. Nine studies reported the ACM and three studies reported MACE on LVLS, as well as three studies reported the ACM, and one study reported MACE on RVLS. The follow-up duration was reported to varying between 1 year and 8.5 years, and the event rate ranged from 10 to 82%. The echocardiographic parameters and prognostic information of the participants are summarized in Table 2.

**Table 2:** Echocardiographic parameters and prognostic information of included studies.

**Study**	**Mean AV area (cm^2^)**	**Mean AV pressure gradient (mmHg)**	**AVmax (m/s)**	**LVEF_pre_**	**LVEF_pos_**	**LV/RV** **LS_pre_**	**LV/RV** **LS_pos_**	**Cut-off** **of LS**	**HR/OR** **(95%CI)**	**Index**	**Ventricular**	**Outcome**	**Event** **rate**	**Hospitalization** **rate (%)**	**Mean or median of follow-up**	**Equipment or platform**
Wani et al. (16)	0.77	41.8	NR	55	57	13.9 ± 4.3	14.8 ± 4.3	NA	0.97 (0.91–1.03)	OR	LV	MACE	35%	30	1 year	GE Vivid E9, E95
Shimoni et al. (17)	0.71	45.9	NR	53.7	53.7	17 ± 5	18.4 ± 4.9	NA	1.130 (1.008–1.127)	HR	LV	ACM	20%	42	1,150 days	GE Echo PAC 202
Ferreira et al. (18)	0.6	57	NR	56.7	NR	13.0 ± 3.8	NR	NA	1.00 (0.88–1.14)	HR	LV	ACM	18%	NR	13.4 months	GE Vivid 9, Vivid E95
								14.8	2.08 (0.59–7.31)							
Omran et al. (19)	0.72	47	NR	51.4	NR	20.0 ± 7.6	19.8 ± 7.8	NA	1.05 (1.01–1.10)	HR	RV	ACM	17%	NR	929 days	NR
Koschutnik et al. (20)	NR	NR	NR	57	NR	22.8 ± 6.9	NR	NA	1.44 (1.03–2.01)	OR	RV	MACE	28%	5	13.7 months	GE Vivid E9,Vivid 7
								20	1.74 (0.91–3.32)							
Vizzardi et al. (21)	NR	51	NR	51	NR	17.6 ± 4.8	NR	NA	1.14 (1.072–1.213)	HR	RV	ACM	82%	NR	8.5 years	GE, Philips, Siemens
Medvedofsky et al. (12)	0.44	49	NR	53	NR	24.6 ± 6.3	26.9 ± 5.8	NA	1.04 (1.01–1.07)	HR	RV	ACM	24%	NR	1 year	Philips iE33; EPIQ 7C
Dahl Pedersen et al. (22)	0.68	41	NR	50.9	NR	12.9 ± 4	NR	12.9	1.52 (0.96–2.40)	HR	LV	ACM	15%	NR	743 days	GE Vivid E 90
Fukui et al. (11)	NR	NR	NR	62.2	NR	18.2 ± 4.1	NR	16	1.36 (0.93–1.99)	HR	LV	ACM	37%	NR	31 months	GE Vivid E95,
Suzuki- et al. (23)	0.65	50	4.5	62	64	15 ± 4.4	16 ± 4.3	NA	1.23 (1.05–1.45)	OR	LV	MACE	10%	NR	591 days	NR
Sato et al. (28)	NR	47	4.37	50	53	12.0 ± 3.7	13.0 ± 3.6	NA	1.05 (1.002–1.11)	HR	LV	ACM	56%	NR	1,345 days	Xcelera, Philips
Kobayashi et al. (15)	0.63	49.6	NR	54	NR	13.0 ± 3.3	NR	15	1.35 (0.24–7.49)	HR	LV	ACM	19%	NR	376 days	GE Vivid E9, E95

AV, aortic valve; LVEF, left ventricular ejection fraction; LS, longitudinal strain; HR, hazard ratio; OR, odds ratio; LV, left ventricular; RV, right ventricular; ACM, all-cause mortality; MACE, major adverse cardiovascular events; NR, Not reported; NA, not applicable.

### Overall analysis

**Figure 2:**
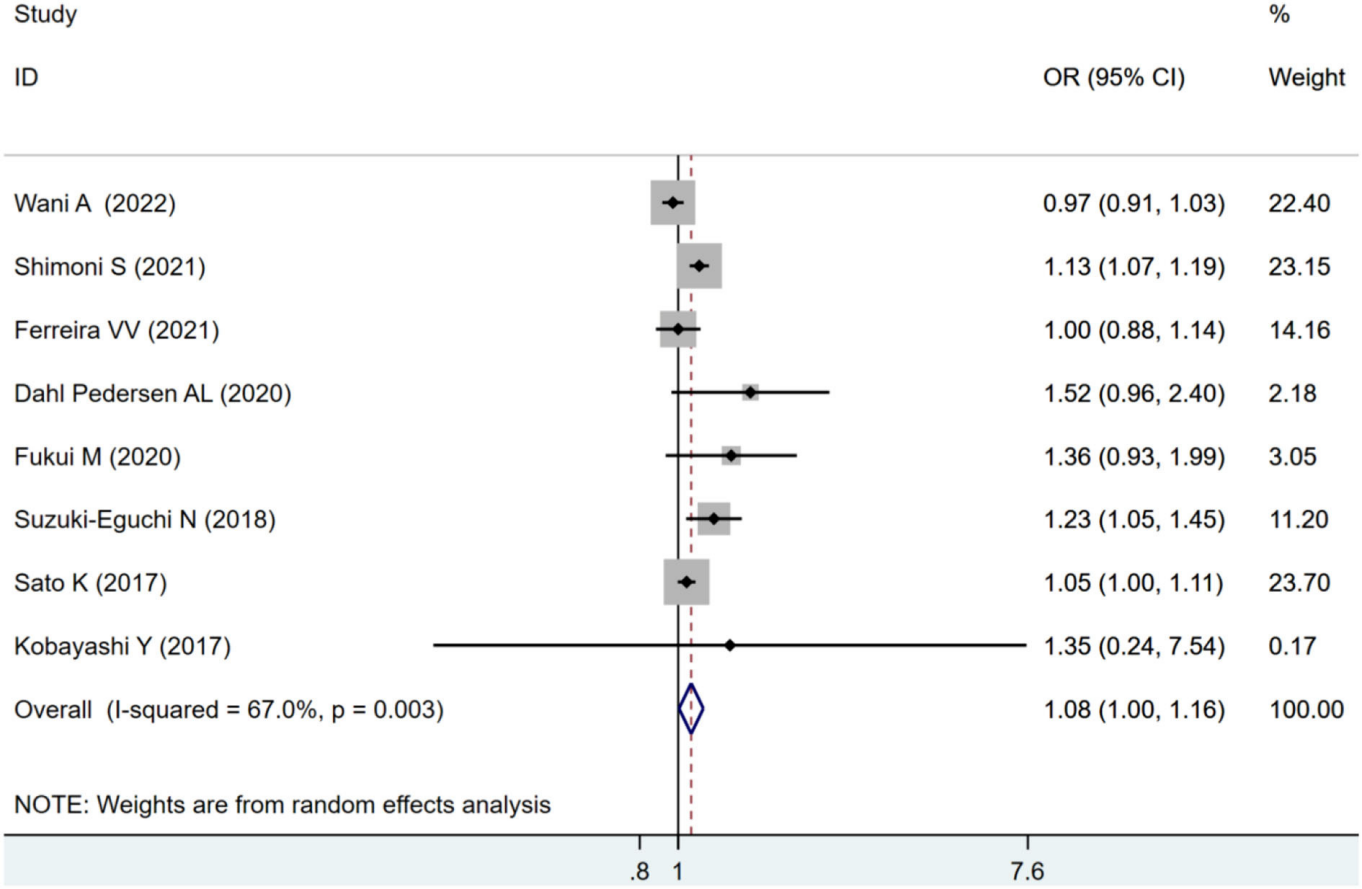
Forest plot demonstrating the association between LVLS and combined ACM and MACE in Patients with AS undergoing TAVR.

Eight studies were eligible for the analysis of the combined endpoint of ACM and MACE. The pooled estimates showed an increased risk of combined ACM and MACE in all included patients with impaired LVLS (OR: 1.08, 95% CI: 1–1.16) with statistically significant heterogeneity (*I*^2^ = 67.%, *p* = 0.037) as seen in Figure 2.

**Figure 3:**
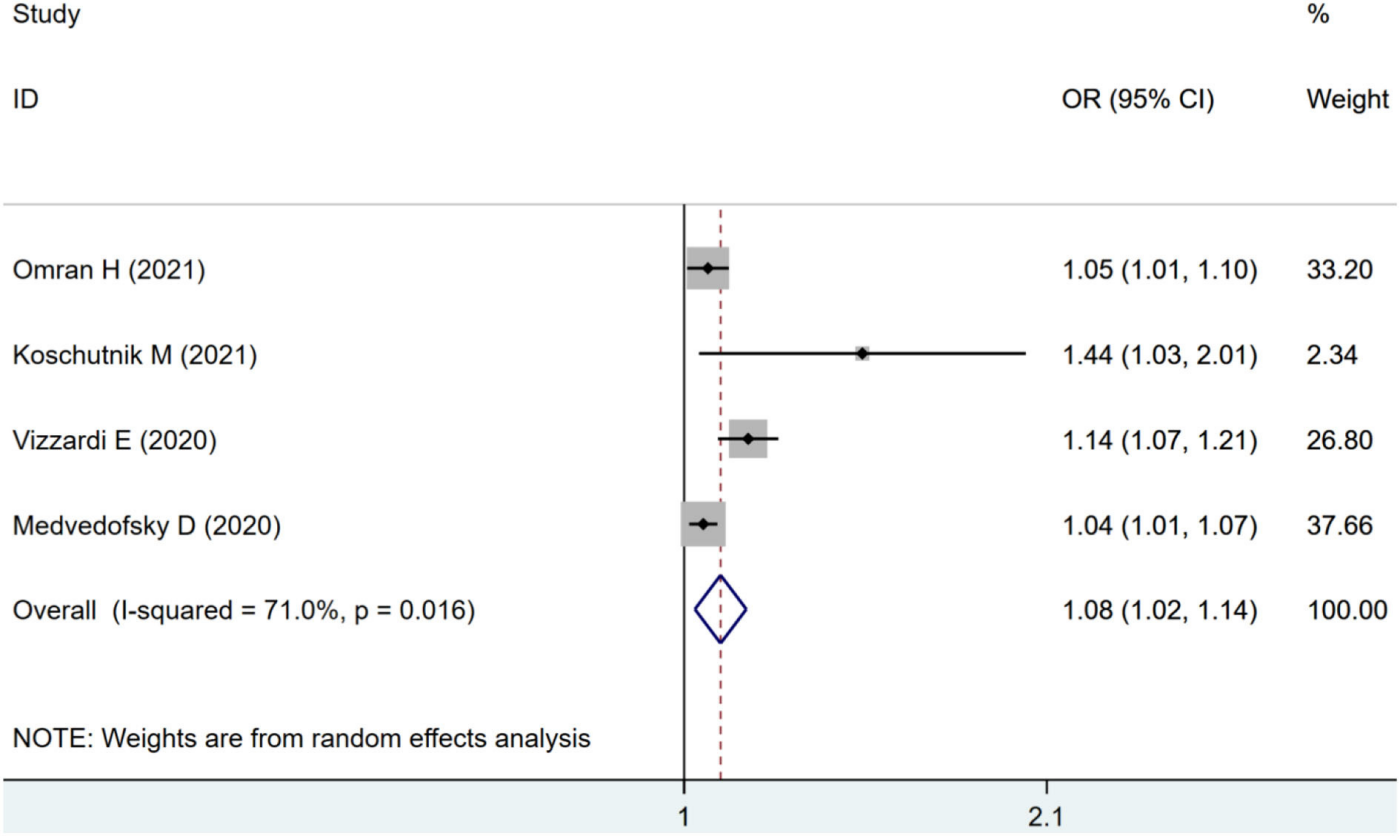
Forest plot demonstrating the association between RVLS and combined ACM and MACE in patients with AS undergoing TAVR.

Four studies were included for RVLS analysis. The pooled estimates also showed an increased risk of combined ACM and MACE in patients with impaired RVLS (OR: 1.08, 95% CI: 1.02–1.14) with statistical heterogeneity (*I*^2^ = 71.0%, *p* < 0.01) as seen in Figure 3.

### Subgroup analysis

**Figure 4:**
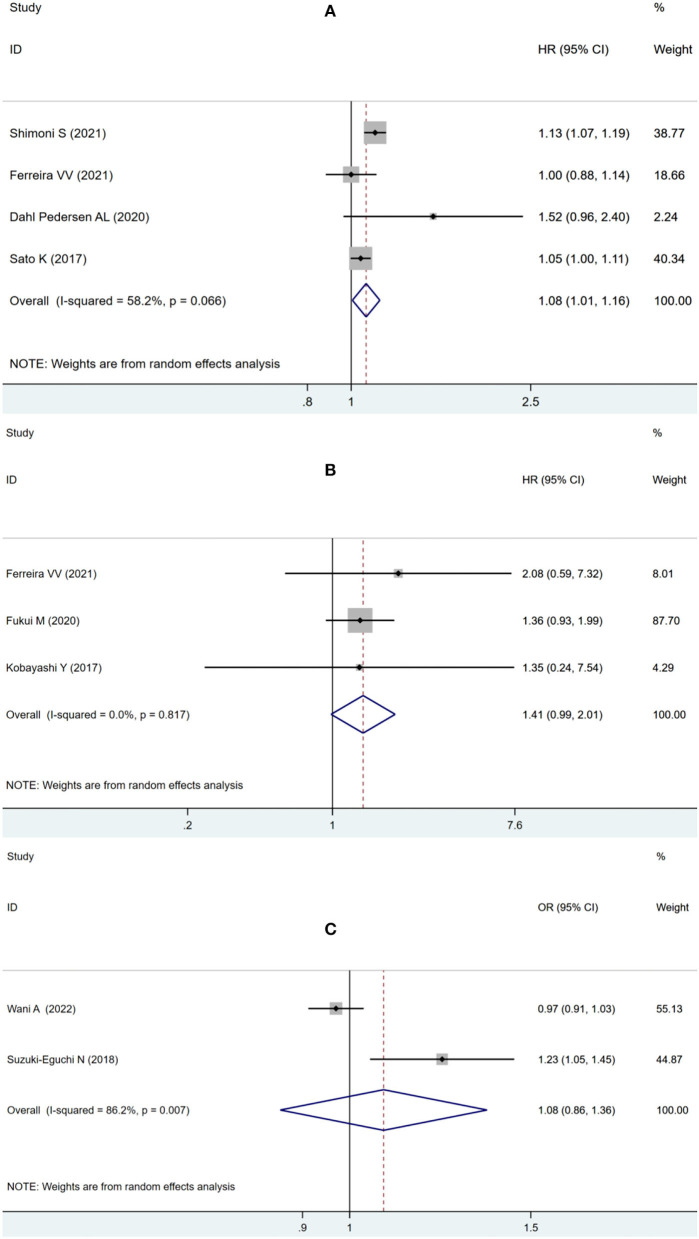
Forest plot demonstrating the association between LVLS as continuous variable **(A)** or binary variable with previously reported cut-offs **(B)** and ACM in patients with AS undergoing TAVR; Forest plot demonstrating the association between LVLS and MACE in patients with AS undergoing TAVR **(C)**.

To assess the possible effect of factors on heterogeneity across studies, a subgroup analysis was performed. According to the adverse outcome of ACM or MACE, the included studies were divided into subgroups and analyzed separately. For ACM subgroup analysis, four studies using LVLS as a continuous variable showed that impaired pooled LVLS significantly increased the risk of ACM (HR: 1.08, 95% CI: 1.01–1.16; *I*^2^ = 58.2%, *p* = 0.032) without evident heterogeneity in Figure 4A. Three studies using LVLS as a binary variable with previously reported cut-offs found that LVLS did not significantly increase the risk of ACM (HR: 1.41, 95% CI: 0.99–2.01; *I*^2^ = 0.0%, *p* = 0.06), with no statistical heterogeneity as seen in Figure 4B.

Two studies were eligible for analyzing the secondary endpoint of MACE, showing no significant increase in MACE risk (OR: 1.08, 95% CI: 0.86–1.36; *I*^2^ = 86.2%, *p* = 0.52) with statistically significant heterogeneity in Figure 4C.

**Figure 5:**
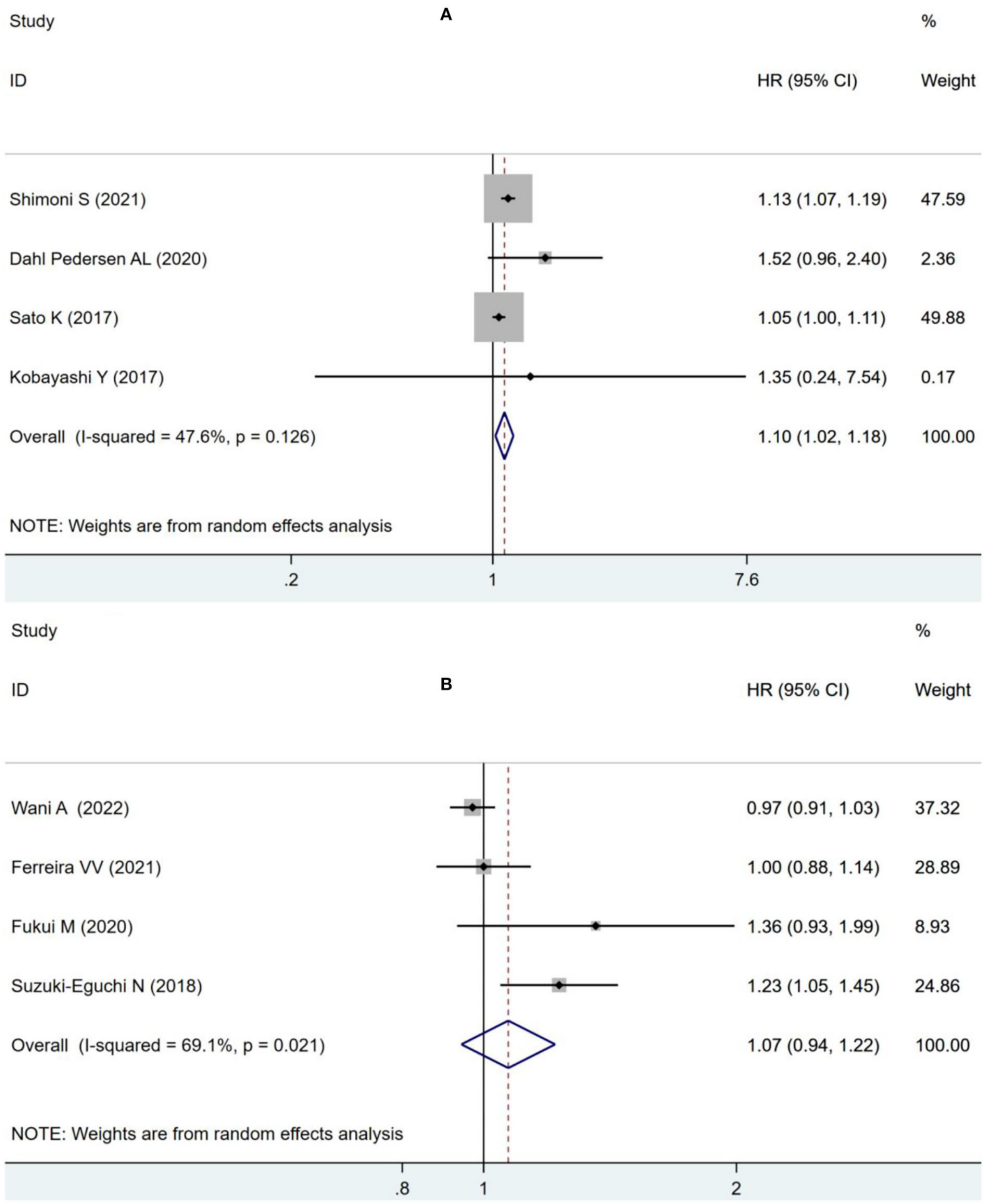
Forest plot demonstrating the association between LVLS and combined ACM and MACE in patients with AS undergoing TAVR in the LVEF <55% group **(A)** and in the LVEF ≥55% group **(B)**.

As suggested by Fukui et al. (11), 55% was used as the cut-off value for LVEF analysis, impaired LVLS significantly increased the risk of combined ACM and MACE (OR: 1.10, 95% CI: 1.02–1.18; *I*^2^ = 47.6%, *p* = 0.011) without statistically significant heterogeneity in the LVEF of <55% group as in Figure 5A, but there was no significant increase in the risk of combined ACM and MACE (OR: 1.07, 95% CI:0.94–1.22; *I*^2^ = 69.1%, *p* = 0.303) with statistical heterogeneity in the group with LVEF exceeding 55% in Figure 5B.

**Figure 6:**
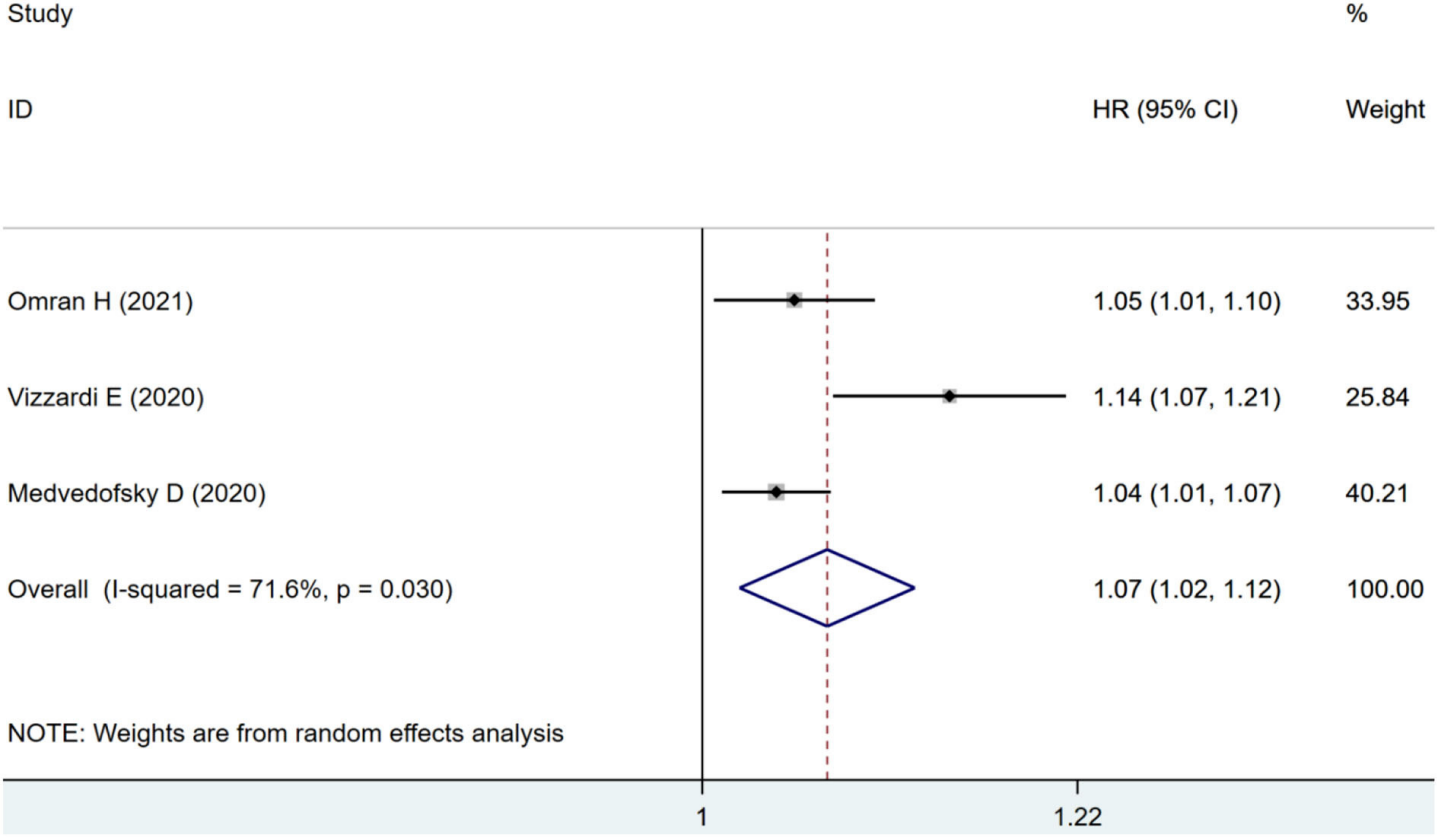
Forest plot demonstrating the association between RVLS and ACM alone in patients with AS undergoing TAVR.

Three studies were included for ACM-alone analysis, where impaired RVLS significantly increased the risk of ACM (HR: 1.07, 95% CI: 1.02–1.12; *I*^2^ = 71.6%, *p* < 0.01) with significant heterogeneity as seen in Figure 6.

### Publication bias and sensitivity analyses

There was a non-significant publication bias for LVLS (*p* for Egger's test = 0.335) and RVLS (*p* for Egger's test = 0.135) in association with combined ACM and MACE in Supplementary Figure S1. A sensitivity analysis was performed to explore the stability of the results. Nonindividual exclusion of studies altered the pooled strain, which supported the robustness of these results in Supplementary Figure S2.

## Discussion

The procedure of TAVR has gained popularity with severe symptomatic AS and has been further developed and trialed in intermediate and low-risk patients (24). As the use of TAVR is extended to younger, lower-risk patients, various overriding issues arise and comprehensively precise assessment becomes the basis for determining successful outcomes for intervention (25). Echocardiography is a simple but useful tool for managing the entire TAVR process, including perioperative assessment of annulus measurements, cardiac function and concomitant valve disease, intraoperative guidance, postoperative assessment of prosthesis function, location, hemodynamic change, and cardiac function recovery (26). The LVEF measured by echocardiography has been considered a main prognostic marker, but it has limitations as it represents the global change in LV volume and cannot reflect subtle myocardial changes.

The LV systolic dysfunction in patients with AS may be due to reversibly increased afterload from the stenotic valve and irreversible intrinsic myopathy. The LV dysfunction detected by strain in patients with AS has been associated with fibrosis, suggesting myocardial impairment from increased afterload. Impaired LVLS has been reported to exacerbate adverse cardiovascular outcomes in patients with asymptomatic AS (27). After TAVR, LVLS improvement was dependent on the degree of fibrosis, LVLS in patients with severe AS might independently predict mortality or an adverse event. But the current results were controversial. Some studies revealed a significant prognostic value of LVLS (17, 28) and concluded baseline LVLS was associated with poor survival (11, 23), but others also found it not associated with a survival benefit (15, 16, 18, 22). This meta-analysis found that the impaired pooled LVLS was associated with an increased risk for combined ACM and MACE. Subgroup analysis found ACM alone was the major factor, but only for LVLS used as the continuous variable, while the pooled result was not significant when different cut-off values, including −14.81% (18), 16% (11), 12.9% (22), and 15% (15), were used. When subgroup analyses were performed based on LVEF using 55% as the cut-off value, the pooled result showed that the impaired LVLS was associated with combined ACM and MACE in the LVEF below 55% group, but not when LVEF exceeds 55%. The pooled results suggested that baseline LVLS provided an independent prognostic value for adverse outcomes, especially for ACM and patients with reduced LVEF, which could be incorporated in TAVR risk stratification models.

There is increasing evidence for the impact of RV dysfunction on mortality after TAVR. The RVLS is also a sensitive marker for detecting subclinical RV dysfunction. Conflicting results have been reported with respect to RVLS association with mortality after TAVR. Omran et al. (19) reported that pre-procedural RVLS significantly predicted long-term all-cause mortality in patients undergoing TAVR; Medvedofsky et al. (12) and Vizzardi et al. (21) also reported a significant association with mortality after TAVR. However, Koschutnik et al. (20) reported no echocardiographic measure, including RVLS was significantly associated with outcome. The results of this meta-analysis were consistent with most results, confirming the better performance of STE in RVLS analysis and its prognostic value in patients undergoing TAVR.

The current study does have some limitations. First, heterogeneity among the studies was observed. The origin of heterogeneity may be due to population characteristics, especially for follow-up time. Given the limited data, meta-regression was not performed. Second, some studies did not provide data directly, but it was obtained from Kaplan-Meier curves, which may increase heterogeneity. Finally, for LVLS analysis, some studies provide the parameter as a continuous variable, and some studies provide binary variables with diverse cutoff values. Well-designed and larger-scale prospective studies are needed to identify LVLS for early recognition of risk stratification in patients with AS undergoing TAVR.

## Conclusion

This meta-analysis demonstrated that ventricular strain including LVLS and RVLS exhibited a substantial prognostic value in ACM or combined ACM and MACE, which could be used as a valid marker for risk stratification in patients with AS undergoing TAVR.

## Data availability statement

The original contributions presented in the study are included in the article/Supplementary material, further inquiries can be directed to the corresponding author.

## Author contributions

YX and WR came up with the idea and supported the work. YX and WB wrote the manuscript. YX and WQ independently screened eligible studies and evaluated the quality of studies. YX and XW independently extracted the baseline and outcome data. The disagreement was resolved by YL and WR. All authors contributed to the article and approved the submitted version.

## Conflict of interest

The authors declare that the research was conducted in the absence of any commercial or financial relationships that could be construed as a potential conflict of interest.

## Publisher's note

All claims expressed in this article are solely those of the authors and do not necessarily represent those of their affiliated organizations, or those of the publisher, the editors and the reviewers. Any product that may be evaluated in this article, or claim that may be made by its manufacturer, is not guaranteed or endorsed by the publisher.
