# Supplementary material for: Prognostic value of ventricular longitudinal strain in patients undergoing transcatheter aortic valve replacement: A systematic review and meta-analysis
Source: Front Cardiovasc Med. 2022 Aug 24;9:965440. doi: 10.3389/fcvm.2022.965440 (PMC9448921; doi:10.3389/fcvm.2022.965440)

**
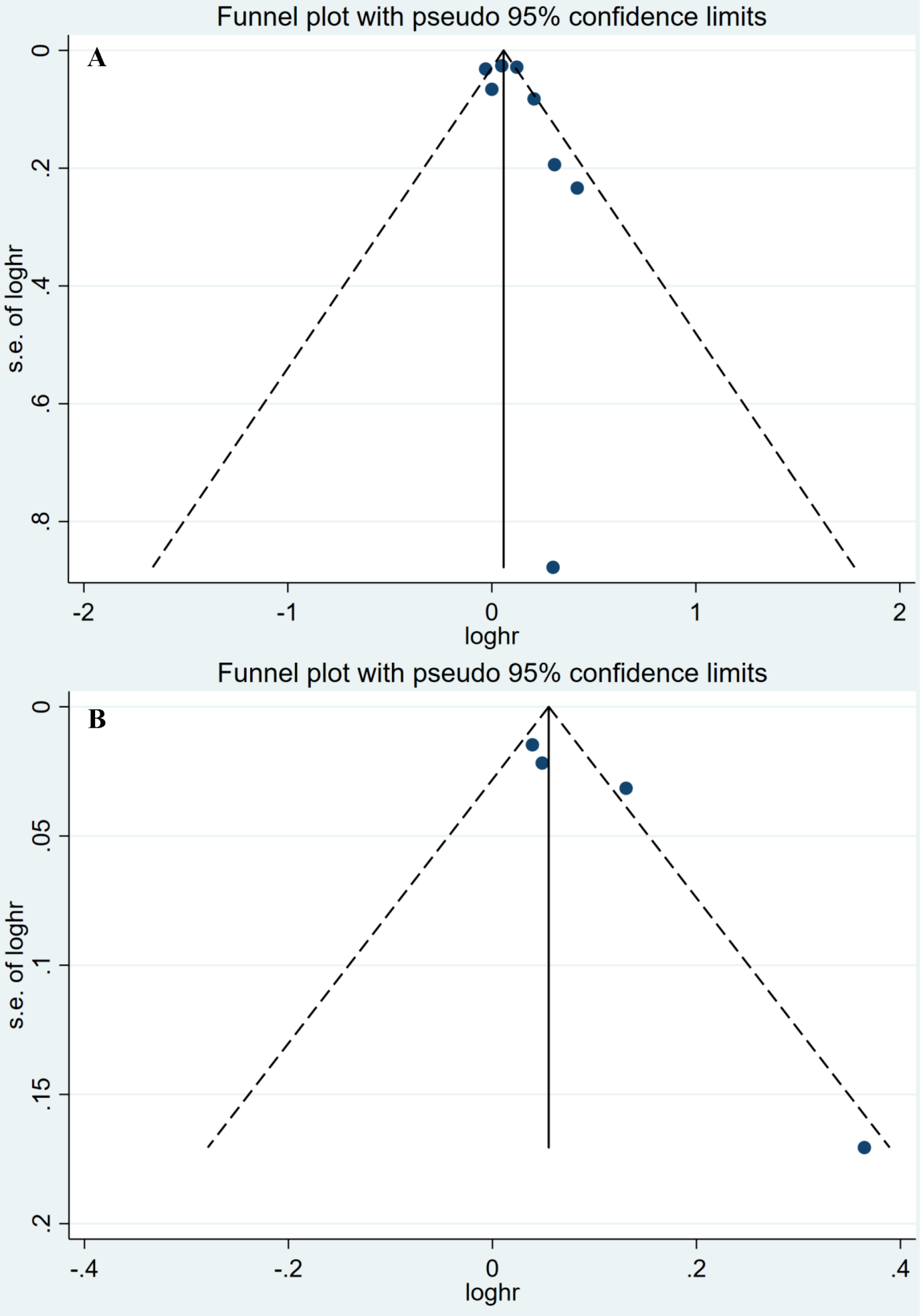
**

**Figure S1** Funnel plot for the association between LVLS (A) and RVLS (B) and combined ACM and MACE in AS patients undergoing TAVR

**Figure S2** Sensitivity analyses using the leave-one-out approach for the association between LVLS (A) and RVLS (B) and combined ACM and MACE in AS patients undergoing TAVR


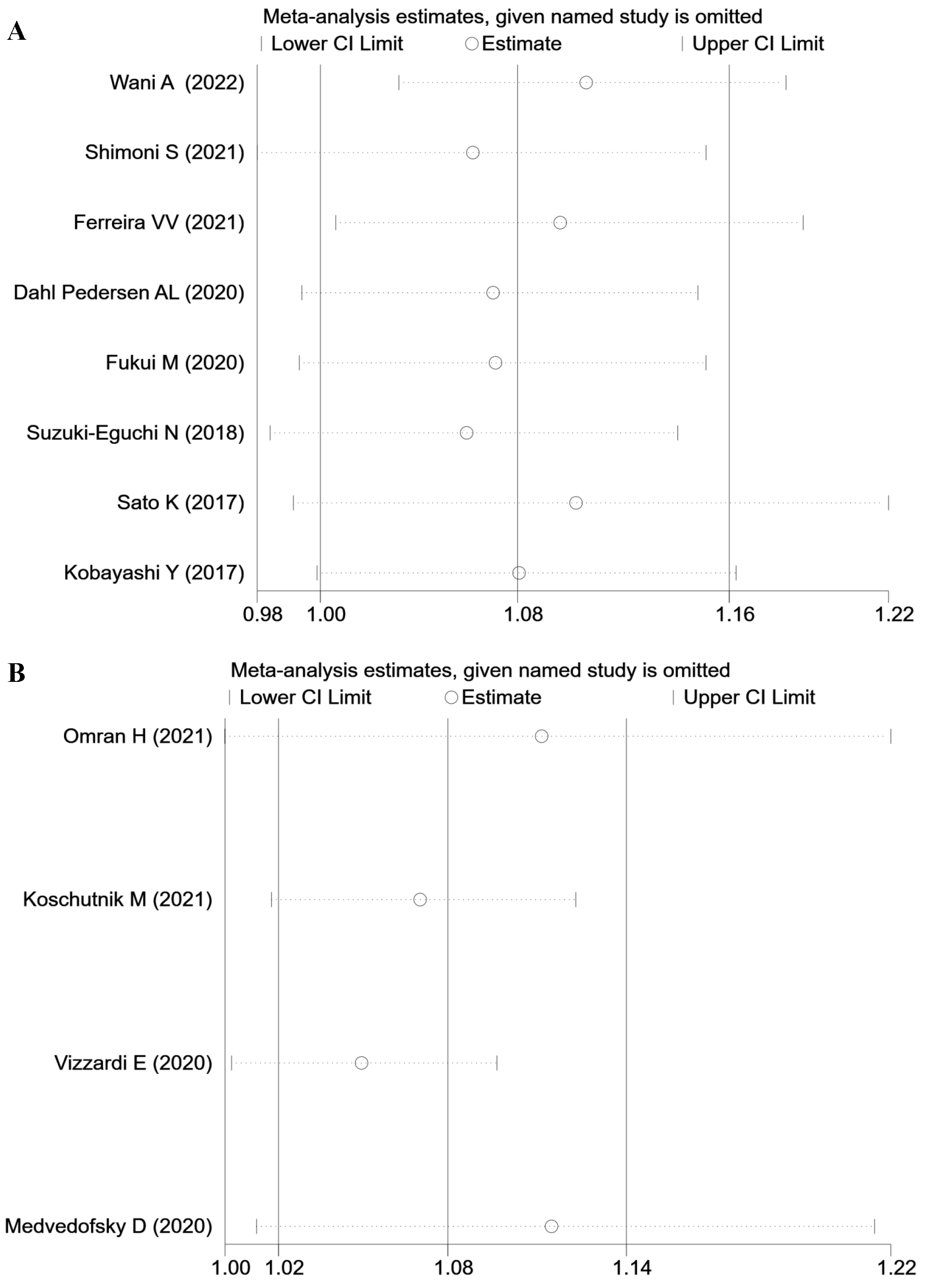

Supplement: Supplementary file 1 [file Data_Sheet_1.docx]
